# Determining the value of preferred goods based on consumer demand in a home-cage based test for mice

**DOI:** 10.3758/s13428-022-01813-8

**Published:** 2022-04-25

**Authors:** Pia Kahnau, Anne Jaap, Kai Diederich, Lorenz Gygax, Juliane Rudeck, Lars Lewejohann

**Affiliations:** 1 German Federal Institute for Risk Assessment (BfR), German Centre for the Protection of Laboratory Animals (Bf3R), Max-Dohrn Str. 8–10, 10589 Berlin, Germany; 2 Animal Husbandry and Ethology, Albrecht Daniel Thaer-Institute of Agricultural and Horticultural Sciences, Faculty of Life Sciences, Humboldt-Universität zu Berlin, Berlin, Germany; 3 Animal Behavior and Laboratory Animal Science, Institute of Animal Welfare, Freie Universität Berlin, Berlin, Germany

**Keywords:** Home-cage, IntelliCage, Group housing, Mice, Consumer demand, Preference test

## Abstract

From the preference of one good over another, the strength of the preference cannot automatically be inferred. While money is the common denominator to assess the value of goods in humans, it appears difficult at first glance to put a price tag on the decisions of laboratory animals. Here we used consumer demand tests to measure how much work female mice expend to obtain access to different liquids. The mice could each choose between two liquids, one of which was free. The amount of work required to access the other liquid, by contrast, increased daily. In this way, the value of the liquid can be determined from a mouse's microeconomic perspective. The unique feature is that our test was carried out in a home-cage based setup. The mice lived in a group but could individually access the test-cage, which was connected to the home-cage via a gate. Thereby the mice were able to perform their task undisturbed by group members and on a self-chosen schedule with minimal influence by the experimenter. Our results show that the maximum number of nosepokes depends on the liquids presented. Mice worked incredibly hard for access to water while a bitter-tasting solution was offered for free whereas they made less nosepokes for sweetened liquids while water was offered for free. The results demonstrate that it is possible to perform automated and home-cage based consumer demand tests in order to ask the mice not only what they like best but also how strong their preference is.

**Supplementary Information:**

The online version contains supplementary material available at 10.3758/s13428-022-01813-8. The raw data of the experiment can be found under: https://zenodo.org/record/6325238#.Yih7FXyZNPY.

## Introduction

In economics, the principle of consumer demand is used to determine the best possible price of a product in order to achieve the highest possible profit. In contrast, the consumer demand test is used with animals as an operant task to assess the value of goods from the animal’s point of view by examining the motivation to obtain or to avoid goods (Cooper, 2004; Lea, 1978). This is achieved by examining how much work animals are willing to perform to obtain goods or to avoid them. In this context, work performance can be equated with the paid price (Lea, 1978). By determining which price is paid for which goods by the animals, it is possible to determine the strength of the preference (Kirkden, Edwards, & Broom, 2003) with a higher price indicating a stronger preference. In addition, demand curves can be used to determine which goods are necessary or luxurious. Therefore, a consumer demand curve is plotted on logarithmic axes depicting the relation of the quantity consumed by the increase of price. Naturally, the amount of consumption is negatively influenced by the price, i.e., with increasing costs the consumption decreases (Dawkins, 1988; Lea, 1978). For necessary goods, which ensure survival or increase fitness, the slope is hardly influenced by the price, the so-called price elasticity is low. However, if the slope is strongly influenced by the price, this indicates that the goods are of little importance or even luxury goods (Cooper, 2004; Dawkins, 1988; Kirkden et al., 2003).

In past studies, animals had to press a lever (Ladewig et al., 2002; Lewejohann & Sachser, 2000) or a switch (Sherwin & Nicol, 1997) in order to receive a reward. The number of lever presses or the energy required to move the switch was used as the equivalent to price. Other obstacles such as a water-filled passageway (Sherwin & Nicol, 1996) or weighted one-way doors (Warburton & Mason, 2003) were also used to make access to the goods more costly.

Laboratory animals were often trained and tested individually in the consumer demand test. Therefore, the animals were either placed in an experimental setup for a few hours per day (Ladewig et al., 2002; Sørensen et al., 2004) or for several days consecutively, using the experimental setup as a home-cage (Manser et al., 1998; Sherwin, 1998; Sherwin & Nicol, 1996, 1997; Timberlake, 1984; Warburton & Nicol, 1998). However, by removing animals from their home-cages and keeping them individually during testing, the animal’s well-being may be negatively affected (Krohn et al., 2006; Manouze et al., 2019). This in turn could have a negative effect on the motivation of the animals to work during the consumer demand test and thus affect the experimental data. Therefore, it seems advantageous to utilize a consumer demand test that allows testing animals that live in groups and in their home-cage with minimum influence of the experimenter. To the best of our knowledge, the first group-housed consumer demand test for mice was developed by Sherwin (Sherwin, 2003, 2004, 2007) who investigated the influence of cage mates on motivation for additional space. Mice were kept in groups, in which only one mouse was trained and thus had access to additional space. As the price increased, the trained mice continued to work for the access to additional space. However, the number of visits and time spent decreased as the price increased. The author argued that additional space seems to be an important resource regardless of the presence of cage mates (Sherwin, 2004).

To test all animals within a social group and to obtain individual data, radio frequency identification (RFID) technology can be used. Past studies showed that the IntelliCage (IC) is a valid home-cage based and automated test setup to analyze activity and learning behavior in mice (Endo et al., 2011; Galsworthy et al., 2005; Kahnau et al., 2021; Krackow et al., 2010; Mechan et al., 2009; Voikar et al., 2018). In addition, the IC allows determining the amount of consumption of liquids and identifying preferences if more than one liquid is presented at the same time.

Animals’ relative preferences for goods have been tested using preference tests. They offer the opportunity to determine which goods are preferred, as the animals themselves can choose between different goods. Especially with regard to animal welfare, it is useful to determine the value of the goods used for improving the living conditions of animals (Dawkins, 1983, 1988, 1990). Preference tests have been widely used in mice, for example, to investigate which bedding and nesting material or enrichment items are preferred (Ago et al., 2002; Banjanin & Mrosovsky, 2000; Chmiel & Noonan, 1996; Freymann et al., 2017; Van Loo et al., 2004, 2005; Patterson-Kane, Harper, & Hunt, 2001; Van De Weerd et al., 1998). There are several different approaches to perform a preference test (Habedank et al., 2018), but usually a binary choice test is performed with two differing goods on offer. Whenever one of these goods is consumed more frequently, or more time is spent with it, it is considered as the preferred one. By combining multiple binary choice tests, it is possible to compare several goods against each other, resulting in a scaling with a defined order. In a previous preference test, we were able to determine a ranking of the liquids (first preference test: 0.2 mM sucrose solution > 10 mM NaCl solution = tap water > 0.4 mM sucrose solution > 10 mM HCl solution, second preference test: almond milk > apple juice > tap water > 10 HCl solution > 3 mM quinine solution) which were also used in this study (the data for this ranking is part of the R package simsalRbim https://talbotsr.com/simsalRbim/index.html). However, such a scaling is just an indicator of the preference under the assumption that all goods are equally accessible. A scaling cannot give information on how much the goods are needed, i.e., a scaling does not determine the strength of the demand for or against a certain good. In order to determine the strength of preference for different liquids, we carried out consumer demand tests using a home-cage based automated setup.

It has already been shown that mice and rats enter a test system, e.g., an automated radial eight-arm maze or a rodent virtual reality (VR) maze, independently from their home-cage through an RFID controlled gate system (Kaupert et al., 2017; Mei et al., 2020; Rivalan et al., 2017; Winter & Schaefers, 2011). In the present study, the setup consisted of a home-cage that was connected via a gate (AnimalGate) to the test-cage (the IC). The IC contained four computerized corners with two liquid dispensers each. Because of the gate, only one mouse was in the IC at a time. This was necessary to allow the individual mice to work undisturbed by group members when accessing the liquids. Otherwise, it would have been possible that the mice interfered with each other directly, for example by pushing each other from the corner of the IC. Given that the home-cage was connected to the test-cage, the mice were basically free to choose when to work for access to the liquids. Since only one mouse could enter the IC at a time, the remaining mice had to wait within the home-cage until the occupant of the IC had left it again. This made it possible to test the mice with minimal influence of the experimenter during their active phase, i.e., when they spontaneously decide to do so and a high level of motivation can be assumed accordingly.

The main objective of this study was to evaluate the feasibility of an automated consumer demand test in a home-cage using the IC system. With this system, we obtained individual data from all mice kept in one social group and we were able to determine different strengths of preferences for different liquids. We expected that the ranking of the liquids would reflect that of the earlier study but provide a more detailed view on the strength of the preference of the tested liquids. Knowing how rewarding or how aversive certain liquids are perceived is a prerequisite for refinement of conditioning experiments. In addition, our group has suggested before that animal welfare can be improved, particularly outside of the actual experiment, by providing rewards (Lewejohann et al., 2020). Finally, taking the mouse's perspective in estimating the strength of preferences of goods will guide future experiments in refining housing and experimental conditions.

## Animals and methods

### Animals and housing conditions

The pre-test of this study with 11 mice was pre-registered in the Animal Study Registry (animalstudyregistry.org, doi:10.17590/asr.0000131). The implementation of the consumer test presented here was based on the experience of the pre-test and was not additionally pre-registered. For the present study, a total of 12 female C57BL/6J mice (Charles River, Sulzfeld, Germany) were used. To ensure maximum genetic and epigenetic independence between individuals, all mice had different mothers and foster mothers. The mice arrived at the institute at an age of 28 to 34 days. During the consumer demand test, the mice were 10 to 19 months old (from November 2019 until August 2020). At the time of testing, the mice were already familiar with the test setup of the consumer demand test because they had participated in the development of a home-cage based cognitive bias test (pre-registered as doi:10.17590/asr.0000121). All mice were handled by the tunnel handling method (Plexiglas, 17.5 cm in length, 4 cm in diameter, for a video tutorial on mouse handling see https://wiki.norecopa.no/index.php/Mouse_handling). Four mice had to be killed due to health issues unrelated to the experiment, and one mouse was found dead (Table 1). The mice were removed from the data analysis of the current run. Even before the experiment, all mice showed fur and whisker trimming behavior, which is commonly found in C57BL/6 mice (Sama et al., 2000).

**Table 1:** Experimental schedule. Four mice had to be killed due to health issues and one mouse was found dead

Run	Abb.	Working corner	Free corner	*n*	Age*	Duration*
1	WQ	Tap water	Quinine hydrochloride dihydrate, 1.3 mM	12	316	64
2	AW	Almond milk, Alnatura, Almond Drink, unsweetened, 1:3 dilution	Tap water	11	387	20
3	WN	Tap water	NaCl, 10 mM	11	427	9
4	S0.4W	Sucrose, 0.4 mM	Tap water	11	444	15
5	WH	Tap water	HCl, 10 mM	10	469	24
6	JW	Apple juice, Sachsenobst Apple juice clear, 1 :3 dilution	Tap water	10	510	24
7	WW	Tap water	Tap water	7	540	9
8	S0.2W	Sucrose, 0.2 mM	Tap water	7	561	10

*n* = number of mice present in the runs and included in data evaluation. Dilutions were made with tap water. *Abb*. abbreviations

* in days

The room temperature and the humidity of the housing/testing room was 22°C ± 3°C and 55% ± 15%, respectively. The dark/light cycle was set to 12/12 hours. Because of the switch from winter to summer time, the light switched on at 7:00 am (MET/CET) in winter months and at 8:00 am (MEST/CEST) in summer months. Half an hour before the light phase, a sunrise was simulated by a wake-up light (Philips HF 3510, 100–240 vac, 50–60 Hz, Philips Consumer Lifestyle B.V. Netherlands). Over 30 min, the light intensity gradually increased until it reached full intensity at 7:00 am or 8:00 am, respectively. The room lights switched on at 7:00 am or 8:00 am and the wake-up light switched off after 1.5 h. The wake-up light was positioned in one corner on the ground of the room with the light shining in the direction of the test setup. The daily visual inspection of the mice was performed between 8:00 and 10:00 am. Once a week, the mice were weighed, inspected for health, and tail-colored (Edding 700, colors: red, black, white, silver, yellow) for individual visual identification. On the same day, the experimental setup including the home-cage was cleaned. All nesting, bedding materials and other enrichment items were replaced, but a small handful of bedding was transferred from the old home-cage to the new home-cage.

For testing within the IC system, it is necessary to implant RFID transponders. Since there were some transponder losses after previous transponder implantations (see supplements), we optimized our procedure. We assumed that the injection site was manipulated by the mice themselves or by group members in such a way that transponder loss occurred. In order to prevent this, the mice received an analgesic (meloxicam 1mg/kg, Meloxidyl by CEVA) the evening before instead of 2 h before the transponder implantation. The duration of the analgesic effect lasted until at least 3 h after implantation, but not until their active phase in the following evening/night. The mice received RFID transponders (Euro ID, FDX-B, ISO 11784/85) under isoflurane anesthesia (induction of anesthesia: 4 l/min 4%; maintenance of anesthesia: 1 l/min 1–2%) at an age of 35 to 41 days. No transponder was lost following this optimized procedure.

**Fig. 1:**
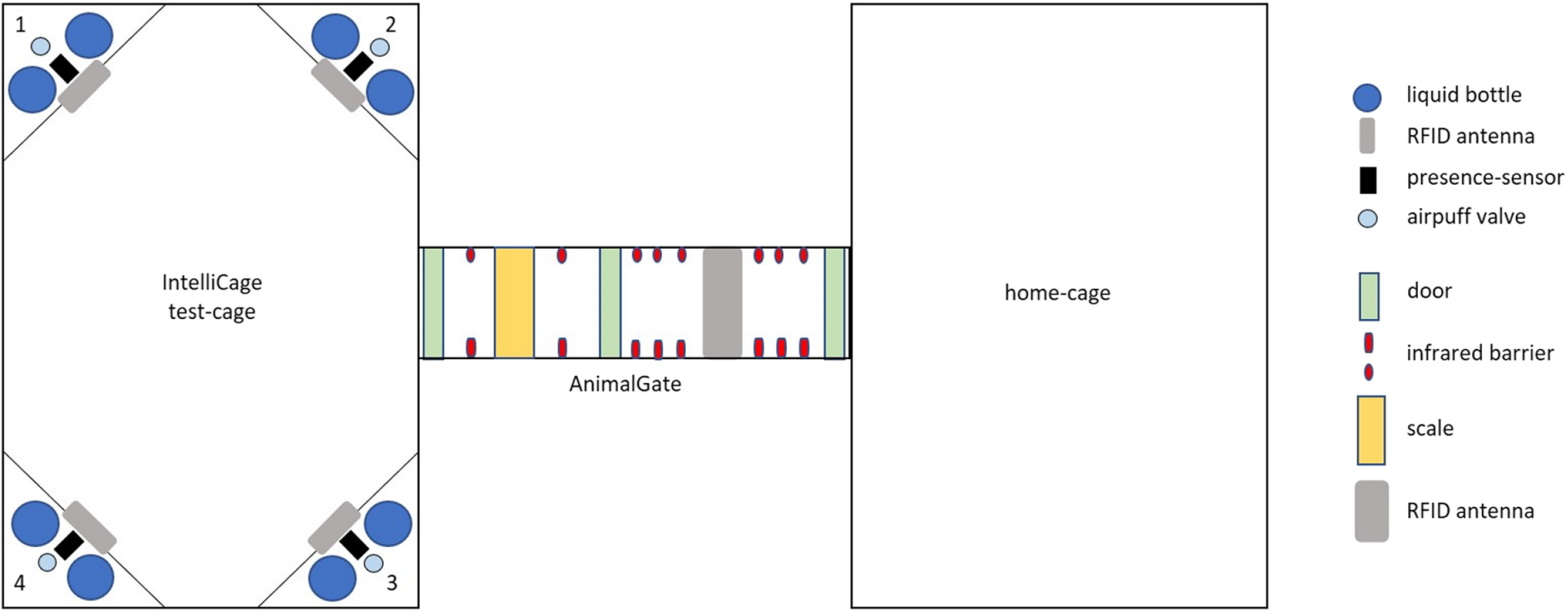
Automated and home-cage-based test setup. The test setup consisted of a test-cage (IntelliCage), a gate (AnimalGate) and a home-cage. The IntelliCage contained bedding but no nesting or food. Within the IntelliCage each of the four corners was equipped with two water dispensers, one radio frequency identification (RFID) antenna, one presence-sensor, and one air-puff valve (air puffs were, however, not used during the consumer demand test). The AnimalGate contained three doors, eight infrared barriers, one scale and one RFID antenna. The home-cage contained bedding, nesting, two shelters, and food which was available *ad libitum*

All 12 mice were housed as one social group in an automated and home-cage based test setup (Fig. 1). The test setup consisted of a home-cage connected to a test-cage (IntelliCage, TSE-Systems, Germany) via a gate (AnimalGate, TSE-Systems, Germany). This allowed the mice to be tested over a long period of time, in their active phase. The home-cage was equipped with 3–4 cm bedding (spruce/fir, 2.5–5 mm, JRS Lignocel FS, Germany), two red houses (“TheMouseHouse”, Tecniplast, Italy), nesting material (eight paper tissues, six cotton rolls, six nesting paper stripes), four wooden bars to chew on, food *ad libitum* (LAS QCDiet, Rod 16, autoclavable, LASvendi, Germany), and one transparent handling tube (4 cm in diameter, 17.5 cm long). In order to gain access to water or the test liquids, the mice had to pass through the gate individually. The gate allowed only one mouse at a time to pass from the home-cage to the test-cage. This was made possible by three doors, one RFID antenna and eight infrared barriers within the gate. The doors remained closed until the mouse returned to the home-cage. The separation allowed the mice to be tested individually and undisturbed by group members. This also implies that the remaining mice in the cage had to wait until the one mouse left the IC again. The gate also contained a scale, which measured the weight of each mouse on each passage to the IC. Each corner of the IC had one RFID antenna and one presence sensor for individual mouse identification. The presence sensor detected changes in temperature. If there was a temperature change within the IC corner and a transponder was detected by the RFID antenna at the same time, this event was counted as a visit. Each corner also comprised two water dispensers. Each dispenser had one lickometer, which measured the number of licks. The access to the liquids could be denied or granted through doors for each dispenser. By performing a nosepoke on the nosepoke-sensors on each door, the doors could be opened by the mice. With the Designer software of the IntelliCage Plus software package the access permissions to certain corners within the IC could be defined for each mouse. In addition, the required number of nosepokes for the access to the liquids could also be defined using the Designer software.

### Consumer demand test

For our consumer demand test, the strength of preference or aversion was tested for eight different liquids in eight sequential “runs”. During single runs, one liquid was offered in both liquid dispensers of one IC corner for which the mice had to make an increasing number of nosepokes every day (working corner). In both liquid dispensers of an adjacent corner the access to a second liquid was offered for the constant price of one nosepoke (free corner). This ensured that the mice did not suffer from thirst and had the possibility to drink at any time. To test the strength of preference, the mice had to work in four runs to gain access to supposedly positive tasting liquids (almond milk, apple juice, two sugar solutions) while at the same time water was offered in the free corner. To test aversion, the mice had to work in three runs to gain access to water while at the same time supposedly bad tasting liquids (bitter, sour, or salty-tasting solutions) were offered in the free corner.

In both working and free corners, the mice were able to drink for 10 s after making the required number of nosepokes. To drink again, the mice first had to leave the corner, re-enter it, and make the required number of nosepokes again. This ensured that while the price of access to the liquids changed, the quantity to be consumed per single access was constant. The working and free corner were the same for all mice but new positions were chosen after each run. This ensured that the new working corner for a new liquid was not used in the previous run.

The new free corner was again adjacent to it. In all runs, the two remaining corners were initially inactive. When a mouse did not execute the required number of nosepokes in the working corner for 2 days in a row, the additional two corners became active while the working and free corner became inactive for this mouse. The mice noticed such a change almost immediately. In the now active corners, the access to water was free (one nosepoke to open the door). This allowed excluding individual mice from the experimental conditions of a given run of the consumer demand test without having to remove them from their social group while the other mice could continue working for an increasing price.

Further on, the names of the single runs (eight runs in total) are abbreviated as follows: The first letter represents the liquid for which the mice had to work in the working corner (Table 1). The second letter represents the liquid that was available in the free corner. If the mice had to work in the working corner for access to, for example, almond milk while water was offered in the free corner, this run is abbreviated as AW. The A represents 3:1 dilution almond milk with tap water, the W represents tap water.

The sequence in which the paired liquids were presented was the same for all mice such that all mice experienced the same odors in the IC. First, the mice had to work for access to water while they had access to a bitter-tasting liquid in the free corner. This run served as training for the operant task (for more information on pre-tests see supplements) and provided data for the first pair of liquids at the same time. The number of required nosepokes to obtain access to the liquid in the working corner was increased daily by one, starting with one nosepoke at day one. For individual mice, each run ended as soon as they did not make the required nosepoke number on two consecutive days. One exception was the WQ run, which was stopped after 64 days, although ten of the 12 mice still made the required number of nosepokes. We decided to stop this run because participation with up to 64 nosepokes let us conclude that the aversion to quinine was very strong. From one run to the next, the mice had to work alternately for obtaining a positive liquid or avoiding a negative liquid (Table 1). Between each run and for 5–8 days on each occasion, all mice had access to water in all four corners by keeping all doors within the IC corners permanently open, therefore, the mice did not have to perform a nosepoke to open the doors. After the last run, the mice had to work one more time for access to water while access to quinine was free. Based on this run, we showed that all mice were still able to perform the operant task. Thus, the decline in motivation to work with rising prices across runs was not due to a nonspecific aging effect. In the last WQ control run, all seven mice that were still in the experiment made up to eight nosepokes for access to water. The run was then stopped (data not shown), because in six out of eight runs more than eight nosepokes were made (see Results section).

### Data analysis

Data analysis and visualization was done with the open-source statistical software R, version 4.0.3 (R Core Team, 2020). Model assumptions were inspected visually by Q-Q plots and by visualizing variance homogeneity of the residuals versus the fitted values. The R package ggplot2 (Wickham, 2016) was used for data visualization.

The setup allowed the mice to enter the IC on their own and one at a time from the home-cage. All other mice had to wait until the IC was free again. The IC occupancy was analyzed based on the time duration during which each mouse was in the IC on each day. For this, the runs WW, WQ, and AW were considered. Runs WQ and AW were chosen to evaluate the influence on IC time of an aversive liquid (quinine) and a preferred liquid (almond milk). Run WW was chosen as a reference because water is a necessary good but should also be neutral compared to quinine and almond milk. The time spent in the IC was used as the outcome in a linear mixed-effects model (R package lme4; Bates et al., 2015). The experimental days were used as a continuous fixed effect (The data for days 53 and 54 of run WQ are missing due to technical problems with the AnimalGate.). The runs (factor reflected by sum-contrast with three levels: WW, WQ, AW) and the interaction of the runs and days were used as additional fixed effects. For the model, the variable day was “centered”. Day seven was chosen as the “middle” of all daily values for centering since observations for all three runs were still made on this day. The runs nested in animals were set as random effects. In addition, the individuality of the daily duration in the IC was evaluated. For this, we calculated the proportion of between-individual variance per total unexplained variance (between- plus within-individual variance) based on the estimated variance components in the model described so far. A confidence interval of this value was calculated using a parametric bootstrap approach with 1000 repetitions (R package lmerTest (Kuznetsova, Brockhoff, & Christensen, 2017) in combination with R package boot (Canty & Ripley, 2021; Davison & Hinkley, 1997)).

In addition to time spent in the IC, we analyzed the number of visits to the IC (IC entries). Since the run WQ ran the longest (with the highest price reached), this run was used for the evaluation. For each day, the sum of IC entries for both the light phase and the dark phase was determined for each mouse. Data were again missing for day 53 and 54 due to the technical problems with the AnimalGate. The logarithm of IC entries was used as the outcome in a linear mixed-effects model (R package nlme; Pinheiro et al., 2020). The experimental days (i.e., price) were used as a continuous fixed effect. The variable phase (factor with two levels: light and dark phase) and the interaction of day and phase were used as additional fixed effects. Sum-contrasts were used for the variable phase. To consider a possible effect of cleaning the setup that was suspected due to a waveform-shape in the number of visits, the variable day since cleaning was added as an additional continuous main effect. The variables day and day since cleaning were normalized for statistical analysis. It was added to the model as an additional fixed effect. The days nested in animals were set as the random effects. For further model assumption inspection, the homogeneity and shape of the residuals versus the variable day since cleaning were visually inspected.

The price paid for access to the liquids were nosepokes which the mice had to make inside the IC working corner. We assumed that as the number of nosepokes increased, the mice had to spend more time (visit duration) within the working corner. Run WQ was selected for analysis because in this run the mice made up to 64 nosepokes for access to water. Only visits in which the required nosepoke number and at least one lick was made, were considered. For the analysis, the visit duration was first determined for each price (required nosepoke number), each visit within the working corner, and each mouse. The logarithm of the visit duration was used as the outcome in a linear mixed-effects model, the price was used as a single fixed effect. Price (is equivalent to the individual test days) nested within the animal was used as the random effects. With this log-transformation, no serious deviations from the assumption could be detected.

The run WW can serve as a kind of control because in both, the working and the free corner, the same liquid was offered. Accordingly, we used the run WW as a reference for further evaluation and we compared the number of drinking events for water in the working corner and water in the free corner specifically in this run. The run WW ran for 9 days. Drinking events were defined as visits in which the mice made the required nosepoke number and drank. The number of these events were used as the outcome in a linear mixed-effects model (R package nlme). In this model, the nine experimental days were defined as days and used as a fixed effect (factor with nine levels). In addition, the type of corner (factor with two levels: working corner versus free corner) and the interaction of type of corner and day were used also as fixed effects. Again, sum-contrasts were used for day and type of corner. The test days nested in animals were set as the random effects.

We examined the maximum price paid by the mice for each liquid in the working corner of each liquid pair. For this, the maximum number of nosepokes they were willing to invest for gaining access was determined for each mouse and for each liquid within the working corner. A survival analysis was used to determine whether the maximum price paid depended on the liquids. This approach allowed for the correct handling of the censored data in the QW trial, i.e., the fact that the mice were still willing to continue working at even higher prices. We calculated this model with the R package survminer (Kassambara, Kosinski, & Biecek, 2020), which implements the cox proportional-hazard model (Coxph) and allowed to reflect the repeated measurement of the mice by defining animal as a “cluster”. The maximum number of nosepokes was evaluated in dependence of the different runs (factor variable with eight levels) as a fixed effect.

Finally, we assessed the elasticity of demand. To do so, we analyzed the slopes of the consumer demand curves defined by the number of drinking events versus price for all liquids within the working corner. For this analysis, a linear mixed-effects model was used again. The log of the number of drinking events (plus 0.5 to allow the inclusion of zeros) was used as the outcome variable. The price (logarithm of the required number of nosepokes), the type of run, and their interaction was used as the fixed effects (using sum-contrasts) and run nested in animal as the random effects. With these log-transformations, we obtained normally distributed residuals. Based on this model, a single demand curve for the liquid in the working corners of each run could be estimated as follows: Y = (I+R) + (SNN + IRNN) * NN, where Y is the estimated (average logarithm of the) number of drinking events, I the intercept, R the main effect of the run, SNN the main effect of the price, IRNN the interaction of the run and the price and NN the price (number of nosepokes).

## Results

### IntelliCage occupancy

**Fig. 2:**
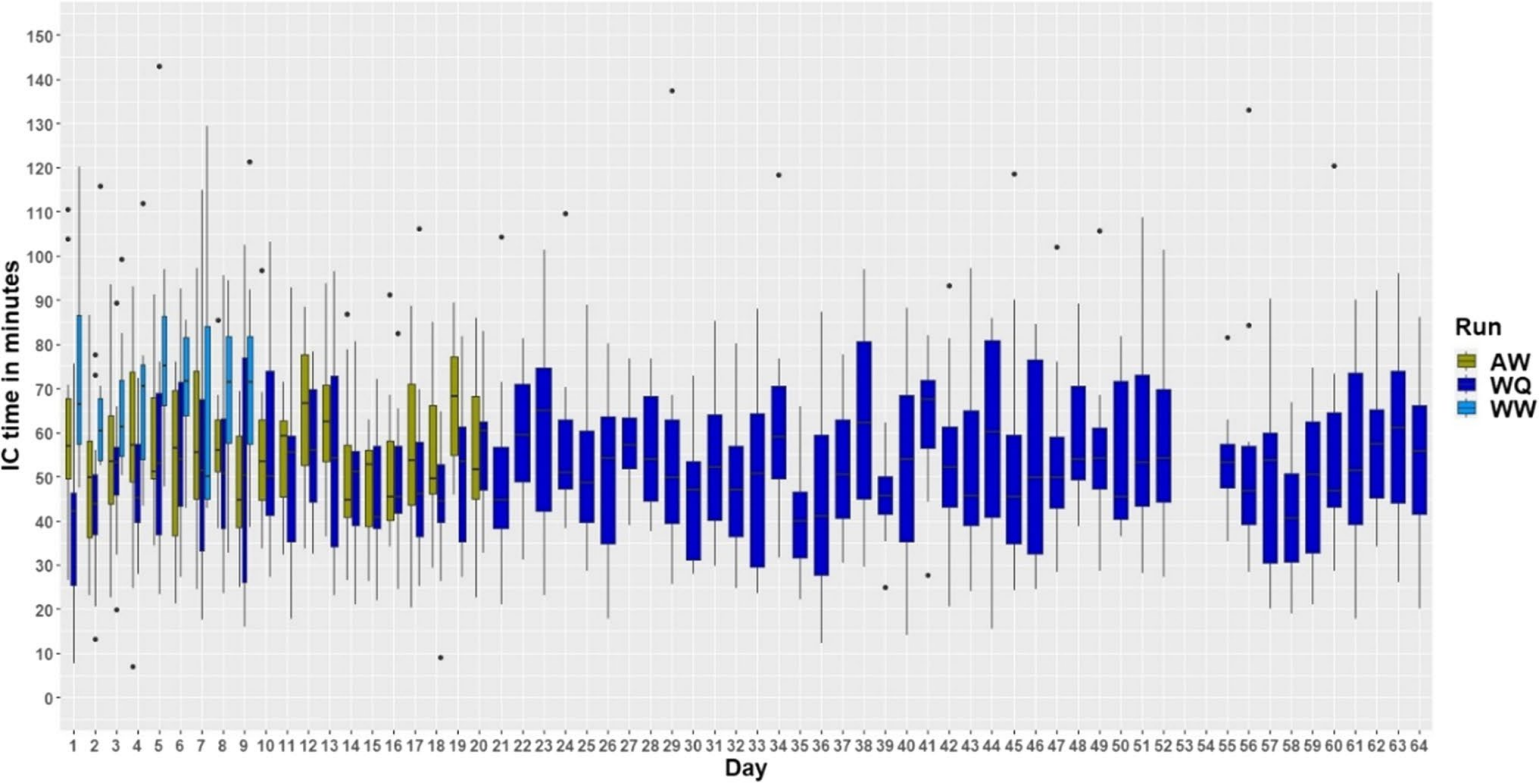
Time the mice spent within the IntelliCage for the runs WW, WQ, and AW (W = water, Q = quinine, A = almond milk). The data for days 53 and 54 of run WQ are missing due to technical problems with the AnimalGate. On the *y*-axis, time spent in the IC by the mice is shown in minutes. The *x*-axis shows the experimental days, which can be equated with the price (number of nosepokes) for the liquids within the working corner

Mice spent the most time in the IC during the run WW (main effect run: *F*_2,12.01_ = 16.73, *p* < 0.0001; Fig. 2) and least during the run WQ. The IC time was not visibly influenced by day (and thus the price to be paid for a liquid; *F*_1,1011_ = 0.03; *p* = 0.85). Moreover, the interaction between day and run had no effect on the time spent in the IC (*F*_2,985.73_ = 0.003; *p* = 1). The proportion of between-animal variability was low at 13.92 % [3.84–25.64 CI] compared to the overall unexplained variability.

**Fig. 3:**
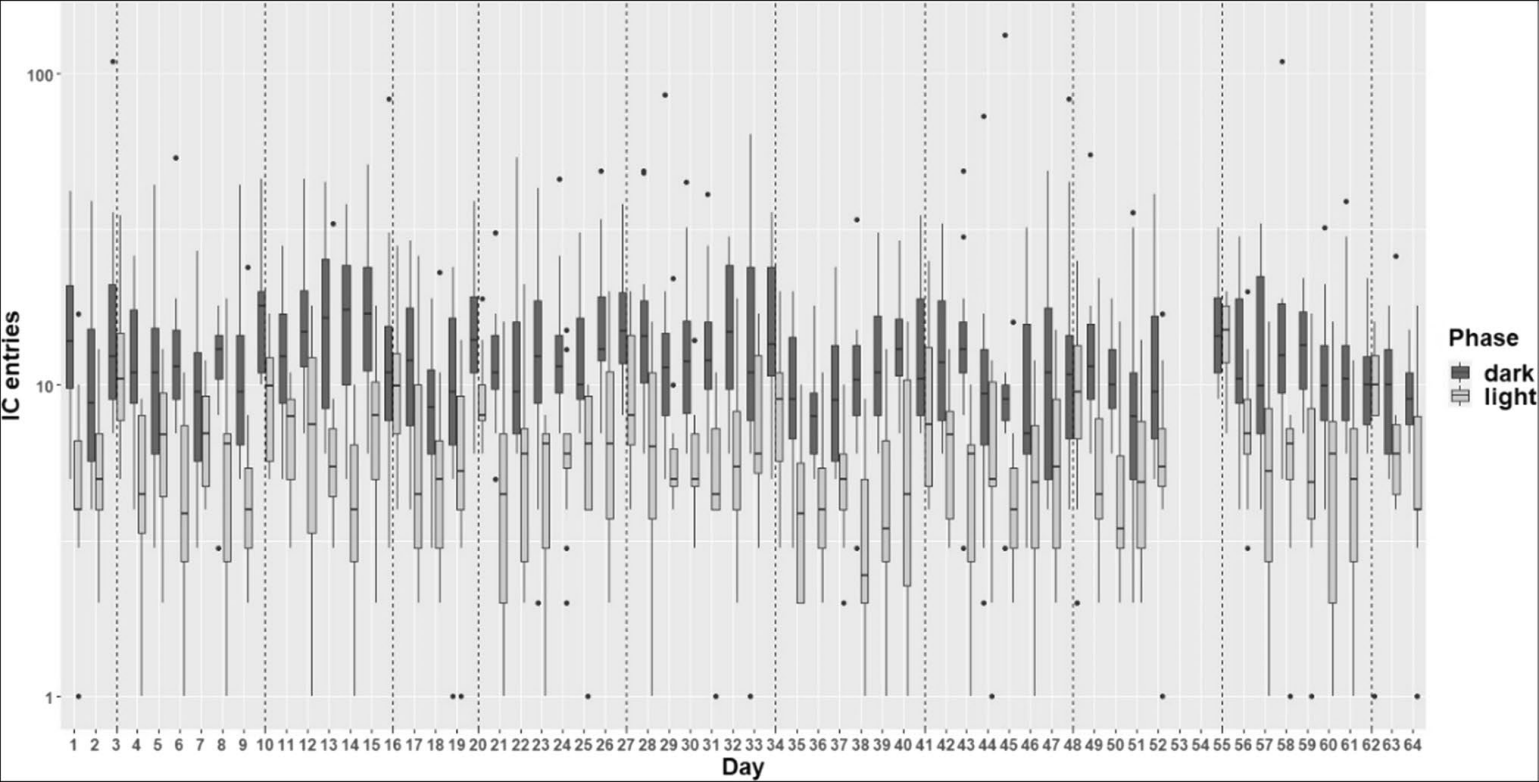
Number of entries the mice made during the run WQ. The data for days 53 and 54 of run WQ are missing due to technical problems with the AnimalGate. On the *y*-axis, the IC entries are shown. The *x*-axis shows the experimental days, which can be equated with the price for the liquids within the working corner. The number of IC entries are shown on a logarithmic scale, while the labels are retained on the original scale. The *dashed lines* mark the days on which the setup was cleaned

On average, the mice visited the IC around 11 times per day (Fig. 3). The mice entered the IC more often during the dark phase than during the light phase (*F*_1,11_ = 34.58; *p* < 0.001). The experimental days and the interaction of day and phase had no influence on the IC entries (day: *F*_1,1452_ = 2.71; *p* = 0.1; day:phase: *F*_1,1452_ = 1.14; *p* = 0.29). The wave-like pattern can be explained by the variable day since cleaning. According to this, the number of entries seemed to decrease after cleaning the setup, especially when comparing the cleaning day to the one that followed (*F*_1,1452_ = 28.51; *p* < 0.001, not shown).

**Fig. 4:**
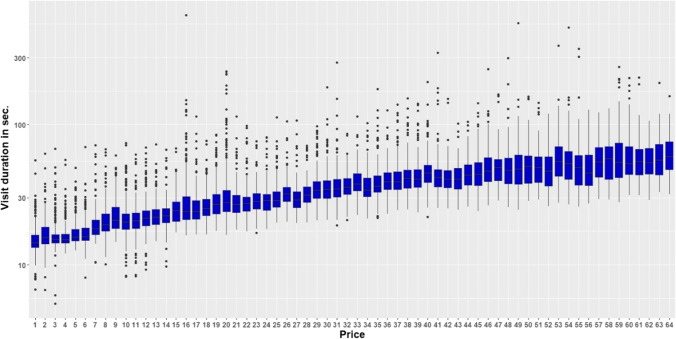
Visit duration in working corner for run WQ (W = water, Q = quinine). On the *y*-axis, the time the mice spent within the working corner is shown. The *x*-axis shows the price the mice had to pay for the access to water. The price can be equated with the experimental days. The visit duration is shown on a logarithmic scale, while the original scale is retained for the axis labels

With increasing price (required nosepoke number) the mice spent more time (visit duration) within the working corner to gain access (effect of price: *F*_63,686_ = 110.49; *p* < 0.0001; Fig. 4). From the figure it is seen that the mice spent around 16 s within the working corner for the price of one nosepoke. It was already around 38 s for the price of 32 nosepokes and around 64 s for the price of 64 nosepokes.

### Comparison of drinking events for run WW

**Fig. 5:**
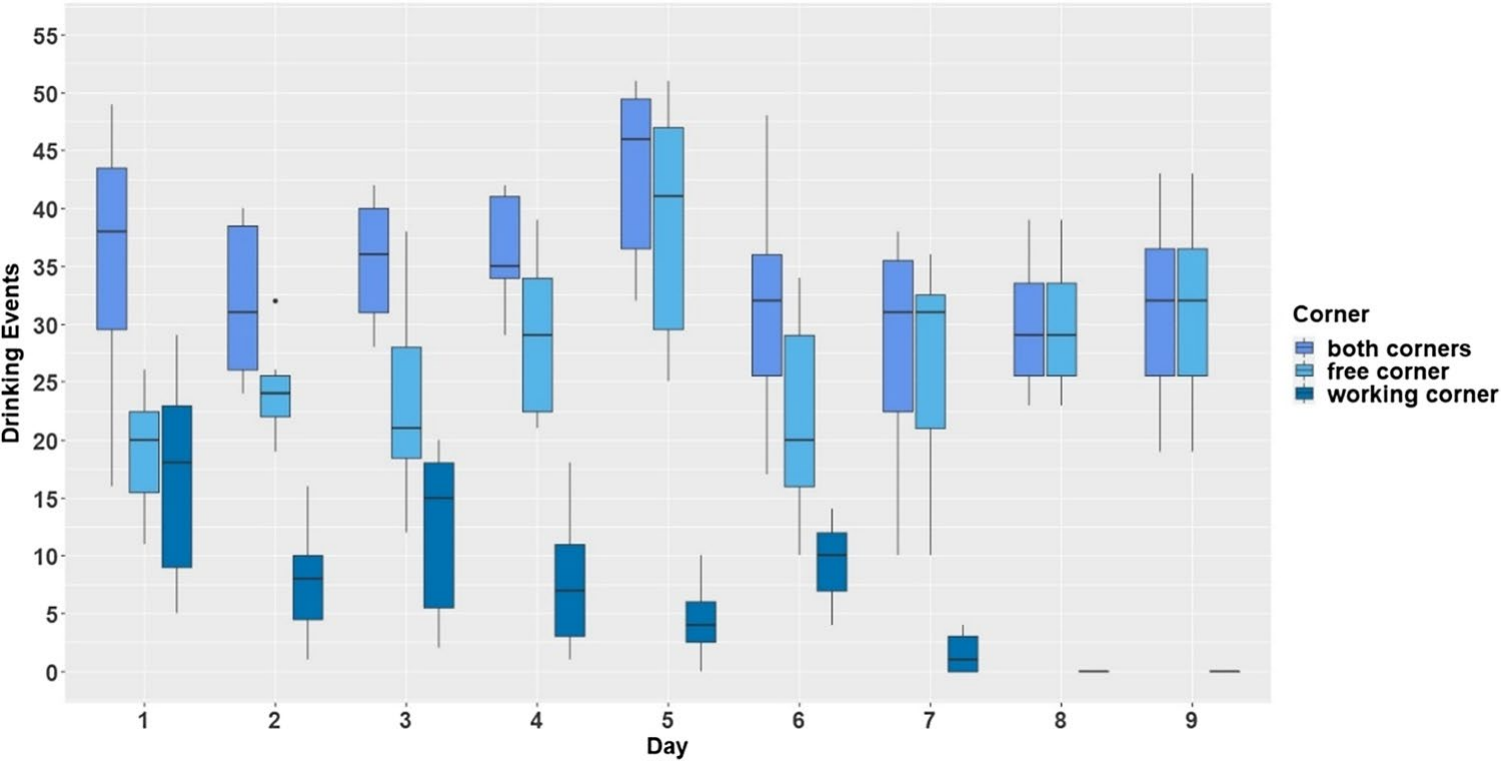
Comparison of drinking events for water in run WW (W = water). The *y*-axis shows the drinking events which the mice made within the working corner and the free corner. The *x*-axis shows the experimental day. The day can be equated with the price the mice had to pay for access to water in the working corner while water within the free corner was available for the price of one nosepoke for all days

The mice made up to seven nosepokes to gain access to water in the working corner while water was available for the price of one nosepoke in the free corner (Fig. 5). On average, there were more drinking events in the free corner than in the working corner during the run WW (main effect corner: *F*_1,54_ = 377.62; *p* < 0.0001). Drinking events in the working corner decreased with increasing price whereas drinking events in the free corner increased (interaction: *F*_8,54_ = 11.2; *p* < 0.0001). In addition, drinking events appeared to decrease slightly with increasing days (main effect day: *F*_8,48_ = 2.07; *p* = 0.058).

### Maximum price paid

**Fig. 6:**
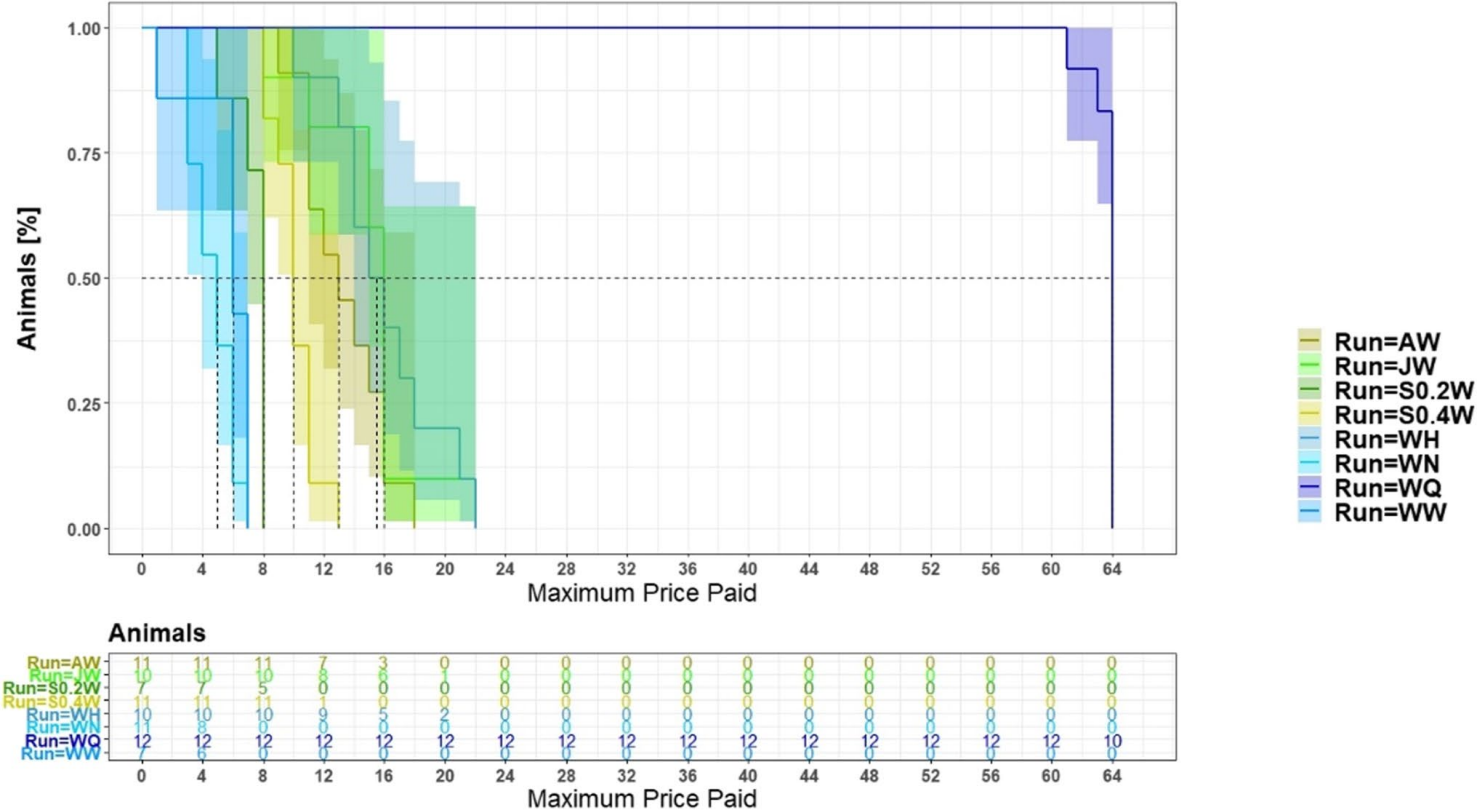
Proportion of mice with specific maximum price paid in the different runs. The highlighted areas are the confidence intervals. The *y*-axis shows the animals which paid the required price. The *x*-axis shows the price the mice had to pay for the access to the liquids. The price is to be equated with the experimental days. W = water, A = almond milk, Q = quinine, N = NaCl, S = sucrose, H = HCl, J = apple juice. For order of runs and sample sizes, see Table 1

The maximum price paid depended on the liquids (Coxph: *p* < 0.0001, Fig. 6). The mice paid the highest price (performed the highest number of required nosepokes) in run WQ. After 64 days, the run was stopped. Ten out of twelve mice made up to 64 nosepokes to gain access to the tap water in the working corner when quinine water was provided in the free corner. To see if this overall influence was caused mainly by run WQ, the data of run WQ were removed for an additional analysis. The influence of the liquid combinations on participation could still be supported (Coxph: *p* < 0.0001).

In run WW and WN (W = water, N = NaCl), the mice paid the lowest maximum price with up to seven nosepokes to gain access to the liquid in the working corner. Mice paid an equally low price for access to a 0.2 mM sucrose solution. For access to a higher concentrated sugar solution (0.4 mM), the mice made up to 13 nosepokes. Up to 18 nosepokes were paid for access to almond milk. In run JW and WH (J = apple juice, W = water, H = HCl), the mice made up to 22 nosepokes to gain access to the liquids in the working corner.

### Consumer demand curve analysis

**Fig. 7:**
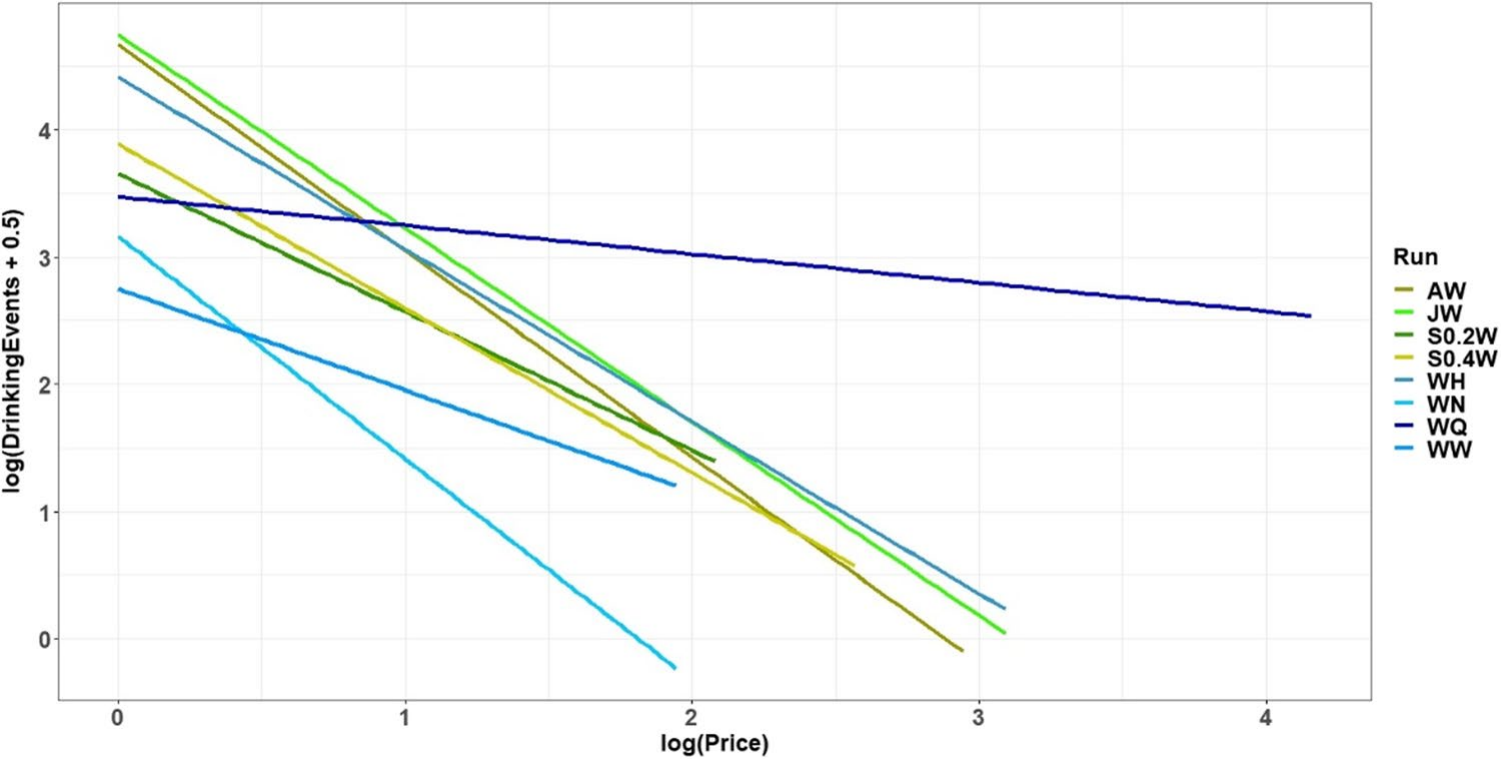
Consumer demand curves. The data are plotted logarithmically. On the *x*-axis are the required nosepoke numbers for the access to the different liquids (price). On the *y*-axis are the values for the number of drinking events for each liquid. The curves end at the maximum number of nosepokes that was reached by any of the mice (A = almond milk, W = water, Q = quinine, N = NaCl, S = sucrose, H = HCl, J = apple juice)

To investigate the willingness of the mice to work for different liquids the slopes of the demand curves were analyzed (Fig. 7, Table 2). The demand curves show the consumed amount (drinking events) on the *y*-axis in relation to the necessary price (required nosepoke number) on the *x*-axis. A more negative slope indicates a lower motivation of the mice to work for the access to the liquids. The comparison of the slopes of all demand curves showed differences compared to the slope of run WW except for run S0.2W. The demand curve of run WQ had the flattest slope. The slopes of run S0.4W, run WH, run JW, and run AW were steeper compared to run WW. The demand curve of run WN had the steepest slope.

**Table 2:** Results of the consumer demand analysis

Run	Slopes	Intercept	*p* slopes	*df*	t	*p* intercept	*df*	t
WN	– 1.75	2.78	0.00	1634	– 4.27	0.24	59	1.19
AW	– 1.62	4.67	0.00	1634	– 4.42	0.00	59	5.75
JW	– 1.52	4.77	< 0.001	1634	– 3.89	0.00	59	5.91
WH	– 1.35	4.43	< 0.01	1634	– 3.01	0.00	59	5.01
S0.4W	– 1.29	3.89	0.01	1634	– 2.56	< 0.01	59	3.36
S0.2W	– 1.09	3.65	0.21	1634	– 1.25	0.02	59	2.34
WW	– 0.79	2.74	reference	1634	– 4.55	reference	1634	9.66
WQ	– 0.23	3.47	< 0.01	1634	3.20	0.02	59	2.32

Run WW served as reference. W = water, Q = quinine, A = almond milk, N = NaCl, S = sucrose, H = HCl, J = apple juice, *df* = degrees of freedom

In addition, the liquid amount consumed for the price of one single nosepoke can be analyzed. The comparison of the amount of drinking events for the price of one nosepoke showed that except for run WN, all runs differed from run WW (Fig. 7, Table 2: Intercept). The smallest amount was consumed in run WW and WN. The largest amount of drinking events was in run JW. The amounts of drinking events of the runs WQ, S0.2W, S0.4W, WH, and run AW were in between of run WW and run JW.

## Discussion

The aim of the present study was to develop an automated and home-cage based test to determine the strength of preferences. For this purpose, we developed a test based on consumer demand theory and determined the strength of preference of mice for different liquids. Our test is using the RFID-based IC system, which makes it possible to test up to 12 mice in one social group over several months while obtaining individual data.

In our setup, the mice were able to independently enter the test-cage (IC) from the home-cage through a gate (AnimalGate). This allowed the mice to work undisturbed by other group members in order to gain access to the liquids. This was necessary because otherwise it would have been possible for group members to gain access to the corner by pulling, pushing, or biting the mouse that was "working". Anecdotally, we can report such behavior in experiments where multiple mice were housed within the IC. It is important to note, however, that the approach of individually channeling mice out of the cage can also result in "wait times" for the other mice in the home-cage. Other studies in which animals were allowed to enter the experimental cage individually have already examined how well individual entry worked, how long this entry lasted, how long habituation took, and how well animals performed in the actual test within the connected test-cage (Kaupert et al., 2017; Mei et al., 2020; Rivalan et al., 2017; Winter & Schaefers, 2011). Since the occupancy of the experimental cage could have an impact on the performance of the other animals, we are also interested in the occupancy of the test-cage. It was found that the time spent in the IC depended on the liquids offered. One might expect that the mice would spend more time in the IC if something positive, such as almond milk, was offered in addition to water. Interestingly, however, the mice spent more time in the IC when water was offered in both, the working and the free corner. The question arose whether some individuals occupied the IC more frequently than others, which would mean that access to the IC would be strongly influenced by these individuals. However, our analysis shows that this does not seem to be the case as we did not detect strong individual variation regarding the overall duration of IC time. This suggests that no single mouse consistently prevented other mice from accessing the liquids by primarily occupying the IC. With increasing required workload, the time spent in the working corner within the IC increased, however, the time spent in the IC per entry was not affected by the price. This suggests that above getting access to the liquids, the stay in the IC is perceived as an opportunity to explore additional space. This is consistent with the results of Sherwin (Sherwin, 2004), who showed that even with increasing price, mice continued to work for access to additional space (although the number of visits and time decreased with increasing price).

On average, the mice spent about 70 min in the IC during the run WW (W = water), which means that the IC is highly used when seven animals are present. Nevertheless, all mice were able to enter the IC and drink. Otherwise, we would have had to offer water separately to the mice that did not drink within 24 h as the IC system automatically warns if a mouse did not drink within 24 h. This was not the case during the entire consumer demand experiment. Through the IC entries, we were also able to show that the mice entered the IC primarily during the dark phase, which is the active phase of laboratory mice. This is in agreement with results of previous home-cage based experiments (Mei et al., 2020; Winter & Schaefers, 2011). However, our mice entered the test-cage more frequently on average (about 11 entries in our study compared to 5.5 entries per day in Winter & Schaefers, 2011). This may be due to the fact that in our study, any liquids were only offered in the test-cage and thereby forcing the mice to enter the test-cage whenever they felt thirsty.

Weekly cleaning of the cages affected the activity in terms of the number of entries made to the IC. The influence of cage changes on activity has already been shown using home-cage based activity measurement (Pernold et al., 2019). However, since all runs lasted for several weeks, we believe it is reasonable to assume that weekly cleaning of the cages did not influence the price the mice were willing to pay. Moreover, in all cases, the mice had two days to rejoin a run if they did not work for 1 day to access the offered liquid. Only after the mice had not worked for the access for two consecutive days, was the run ended for them. For differently structured experiments, however, the changes in daily activity related to cage cleaning may be of importance. Therefore, we recommend for home-cage based experimental designs to cautiously consider effects of intervention by the experimenter (i.e., cage cleaning, health inspections).

To assess the price paid by the mice, the amount of time the mice spent in the working corner was examined for the run WQ (W = water, Q = quinine). As the price increased, the mice also spent more time in the working corner. This shows that in addition to the movement expended to execute the nosepokes, work time can also be considered as another price component. To our knowledge, it had not been considered in recent consumer demand experiments how much time the animals had to spend on the work. In our experiment, this factor was of additional importance, as it possibly affects separating/singulating the mice into the test-cage, since only one mouse can be in the IC at a time. Nevertheless, as stated above, the overall occupation time of the IC was not affected by the increased amount of time spent in the corner.

Regarding home-cage based testing, it can be summarized that it is a well-functioning system for female mice to obtain individual data despite group housing. It should be noted, however, that male mice show much more conspicuous dominance behavior (Van Loo et al., 2004). However, we have recently shown that groups of 12 male mice of the strain C57Bl/6J can be housed without notable aggressive behavior in the IC for a very long time (Kahnau et al., 2021). To validate the suitability of our proposed home-cage based system for male mice, the same experimental design should be performed with males in a future study.

For the evaluation of motivation (the strength of preference) for or against a certain good, the maximum price the animals are willing to pay can be taken into account and compared for different goods (Kirkden et al., 2003). It has been stated that in order to compare the demand of different goods with each other, a benchmark value with a necessary good such as food should be generated (Cooper, 2004; Dawkins, 1983). We believe that our dataset indeed can be used as such a benchmark as it provides information how water as a necessity relates to different liquids either tasting better or worse. However, it should be noted that different wants for goods can interact with each other. Therefore, it is important to compare different motivations for wants in a meaningful way (Gygax, 2017) in order to obtain a suitable benchmark. Since water was offered in all runs in our study, the run WW, in which water was offered in both the working and free corner, was chosen as a reference. Although the drinking events in the free corner were higher than in the working corner, over 50% of the mice were willing to make seven nosepokes for getting access to water in the working corner. As the price exceeded seven nosepokes, all mice refused to work for water while they could have it for free in the other corner. The run WW was deliberately conducted at the end of the whole experiment because this enabled testing how willing the mice were to make nosepokes for water even after long experimental duration. However, the mice might have developed a habit to do nosepokes but this formed routine could not be related to the corner itself, as the position for the working and free corner within the test-cage were changed after each run. Therefore, we assume that the run WW can be used as a valid benchmark in our consumer demand experiment.

The phenomenon to perform an operant task in order to receive a reward in spite of the same reward being additionally available for free, is known as "contrafreeloading" (Jensen, 1963). Past studies showed that different species worked for access to food even while food was freely available. There seem to be individual differences as well as genetic influences (Jensen, Schütz, & Lindqvist, 2002; Lindqvist & Jensen, 2009). The willingness to work voluntarily despite not being obliged to do so, can be seen as an indication that work in itself has rewarding properties. This is especially true for laboratory animals, which usually live under conditions that limit their experience (Lewejohann et al., 2020). While wild mice spend time for foraging behavior, nest building or breeding, laboratory mice have a lot of time on their hands as there is not much else to do while they are “waiting" for the next experiment. Consequently, the determined boundary of seven nosepokes, which were performed as contrafreeloading, might serve as a benchmark in our artificial economy. This benchmark would indicate the maximum number of nosepokes mice are willing to perform due to their lack of alternative activities. This is also supported by the finding that the mice drank less water overall from the seventh day onwards during the run WW. If the "work" becomes too "expensive", a smaller amount of water is drunk, i.e., the water intake in the working corner is added to the basic requirement during contrafreeloading.

In the analysis of the maximum paid price, liquids for which the mice performed more than these seven nosepokes might be considered as having a higher priority than work in itself. In our study, this is true for all liquid combinations except water compared with NaCl, because the mice made only up to seven nosepokes in the run WN. The aversion to a NaCl concentration of 10 mM did not seem to be very strong, because the mice were not willing to work more not to drink this. The run S0.2W (0.2 mM sucrose concentration) also did only differ by one additional nosepoke with regard to the maximum paid price compared to working for water in both corners. Refusing to work more than eight nosepokes for a 0.2 mM sucrose concentration indicates a low strength of preference for mildly sweetened water.

To determine which goods are necessities or luxuries, a consumer demand curve can be plotted on logarithmic scales depicting the relation of the quantity consumed and the increase in price. A demand curve with low elasticity (the slope is hardly influenced by price) indicates necessary goods. However, a demand curve with high elasticity (slope strongly influenced by price) indicates luxury goods. It is important to note that if the quantity of goods that can be acquired per "purchase" is not constant, the price itself changes in terms of inflation. Therefore, Kirkden and Pajor note that the quantity of the good to be consumed should remain the same at any price to avoid other factors, such as time, influence the animal’s motivation (Kirkden & Pajor, 2006). Accordingly, the price of access to the liquids changed in our study, but the time the mice were able to drink remained constant (10 s). In our study, all consumer demand curves of the different runs were compared to the run in which water was offered in the working and free corner (run WW). The slope of the consumer demand curve of run S0.2W (S 0.2 = 0.2 mM sucrose) did not differ from the slope of the consumer demand curve of run WW. The slope of the consumer demand curve of run WN (N = NaCl) was even greater than the slope of run WW. This indicated that the demand curve of WN was more influenced by the price (high price elasticity) and indicated a low motivation to work for access to water while access to 10 mM NaCl concentration was available for the price of only one nosepoke. In contrast to this, the motivation to work for water while access to quinine was available for the price of one nosepoke seemed to be very high. Accordingly, the price elasticity in the run WQ is the lowest.

In human microeconomics, consumer demand theory is based upon the amount of disposable income that can be spent on different goods in the market. In our experimental setup, however, there is only one good that a mouse can work for at a time. As a consequence, the value of the liquids might be overrated due to this methodological constraint. However, our approach allows us to directly relate the worth of the goods on the market to the workload the mice are willing to pay for access. Nevertheless, in our consumer demand test, all curves, except run WQ, seem to show a high price elasticity. This could be due to the fact that in all runs an alternative was offered in the free corner and thus the need to work was less strong. Therefore, it is appropriate to determine the motivation for getting different liquids, additionally by analyzing the maximum price paid.

To evaluate the motivation to obtain goods it is essential to ensure that the animals have indeed learned the operant task in order to exclude misinterpretation (Dawkins, 1990; Rutter & Duncan, 1992), which was also shown by our results of the pre-test (data shown in the supplements). In addition, the time point when the test is performed should be considered. Acosta and colleagues showed that for mice, which are nocturnal, the motivation to work for food is higher at night than during the day (Acosta et al., 2020). Also in our study, mice entered the test-cage more frequently in the dark phase than in the light phase. Thus, considering the time point of performance is crucial for avoiding misinterpretation of the demand curve or maximum paid price. Basically, the maximum amount of work the mice paid for the access to the different liquids tested can serve as a simple benchmark for future studies.

Home-cage based test setups have proven to be useful tools to overcome issues such as day/night rhythm or experimenter influence (reviewed in Richardson, 2012 and Voikar & Gaburro, 2020). In some experiments, it is necessary to keep the animals separately to obtain individual data. However, as mice are social animals, single-housing should be avoided if possible. So far, there are not many systems that allow testing mice in groups while obtaining individual data (some examples reviewed in Voikar & Gaburro, 2020). For the development of such automated and home-cage based systems, it is possible to use a gate to connect the home-cage to the test-cage (Winter & Schaefers, 2011). For example, Mei and colleagues used such a gate to connect a home-cage to an eight-arm radial maze (Mei et al., 2020). We also demonstrated that the mice were able to independently enter the IC several times a day to access the obtained liquids in the IC.

In our study, we developed an automated and home-cage based test setup by using the IC system, in which the mice were tested over several months, in their social group, familiar environment and during their active phase. As a result, influences such as the day/night rhythm or the experimenter could be minimized. In addition, each mouse could work undisturbed by cage mates for access to the liquids. This had the benefit that individual mice took the time they needed to pay the required price. Especially in run WQ, in which the mice made up to 64 nosepokes, the execution of the required nosepoke number took some time (as the duration increased with increasing price), the interruption-free environment will probably have facilitated the task. The mice were able to perform the operant task repeatedly, which reflected the motivation of the animal. By connecting the test-cage to the home-cage, the mice were free to choose if and when to do the required nosepoke number in the working corner (unless a cage mate was currently occupying the IC). Furthermore, by having the mice work again for access to water while they had free access to a bitter-tasting liquid in the free corner after the last run, we were able to show that even in old age the learning task was successfully performed by the mice (see supplement S1).

The consumer demand curves and number of animals that paid the corresponding price showed that the motivation was different and depended on the liquid. A previously conducted preference test already showed that almond milk and apple juice were preferred and the sour- and bitter-tasting liquids were least preferred. With the consumer demand test, these preferences were confirmed. Furthermore, we can show that the aversion to the bitter-tasting liquid is markedly stronger compared to the sour solution but also compared to the preference to almond milk or apple juice. The results may be used to select suitable stimuli for operant tasks in order to optimize learning behavior. We also found that operant conditioning was highly facilitated when the mice had to work for water to avoid drinking a bitter-tasting solution. The experience gained in that trial could then easily be transferred to working for rewarding liquids in consecutive trials. In addition, the data might be of interest for the husbandry of laboratory mice where acidified water is quite common due to the fact that acidification is used to keep water as pathogen-free as possible. Our data indicate a relatively strong aversion to a 10 mM HCl solution (pH = 2.5). This could serve as a reference for acidifying water to facilitate fluid intake by the animals.

With our test setup, it is currently only possible to examine different liquids. However, this setup is a proof of concept for future studies for example in order to optimize housing conditions. For example, when letting the animals choose between different enrichment items, it would be important to also know how strong their preference is. Therefore, we are in the process of developing an automated and home-cage based test setup, combining the mouse positioning surveillance system (MoPSS, Habedank et al., 2021) with the knowledge gained from this study. In our view, one major lesson learned is to let the mice enter the test-cage independently and thus work undisturbed in it. We showed that this experiment could be carried out without a large amount of personnel time (approx. 30–40 min daily (checking the animals and their drinking behavior, preparing and changing liquids, checking the apparatus, approx. 1.5 h weekly cleaning of the setup). In addition, a certain basic technical understanding is advantageous as well as a daily control of the data to check whether the setup is running properly. This knowledge will give us the opportunity to integrate the animals' point of view in the husbandry as well as the experiments themselves and a more comprehensive understanding of the needs and wants of our laboratory mice.

## Supplementary Information

ESM 1 (DOCX 317 kb)
