# Supplementary material for: Determining the value of preferred goods based on consumer demand in a home-cage based test for mice
Source: Behav Res Methods. 2022 Apr 25;55(2):751–66. doi: 10.3758/s13428-022-01813-8 (PMC10027648; doi:10.3758/s13428-022-01813-8)
Supplement: Supplementary file 1 — (DOCX 317 kb) [file 13428_2022_1813_MOESM1_ESM.docx]

Supplements of “Determining the value of preferred goods based on consumer demand in a home-cage based test for mice”

Repetition of run WQ

The mice had to work again for access to water in the working corner (after the last run S0.2W) to avoid drinking a bitter-tasting quinine solution in the working corner (run WQ2). This run was ended after eight days and performed to assess whether the mice were still willing to work for access to water, or whether (perhaps due to age) their willingness had decreased. Run WQ2 was compared to the first run in which the mice had to work for water in the working corner to avoid drinking quinine in the free corner (run WQ).

For comparison, the drinking events of the first eight days and those of the remaining seven animals in run WQ2 were evaluated. The number of the drinking events were used as the outcome in a linear mixed-effects model. In this model, the eight required nosepoke numbers were used as a fixed effect (factor with eight levels). In addition, the type of run (factor with two levels: WQ2 versus WQ) and the interaction of type of run and required nosepoke number were used also as fixed effects. To allow for the interpretation of the p-values of the main effects even with an interaction in the mode, we used sum-contrasts for run and type of corner. The runs nested in animals were set as a random effect. Model assumptions were inspected visually by Q-Q plots and by considering the variance homogeneity of the residuals versus the fitted model.

Data analysis showed that more drinking occurred over the eight experimental days (required nosepoke number) in run WQ2 (Fig. S1, linear mixed-effects model: num. DF = 1, den. DF = 48, F = 66.69, p < 0.0001). The price also had an influence on drinking events. With increasing price (required nosepoke number) the number of drinking events decreased (num. DF = 7, den. DF = 42, F = 4.36, p < 0.001). The interaction of price and run influenced the number of drinking events, too (num. DF = 7, den. DF = 48, F = 2.52, p = 0.03). Thus, it could be shown that the mice were still willing to work for access to water.


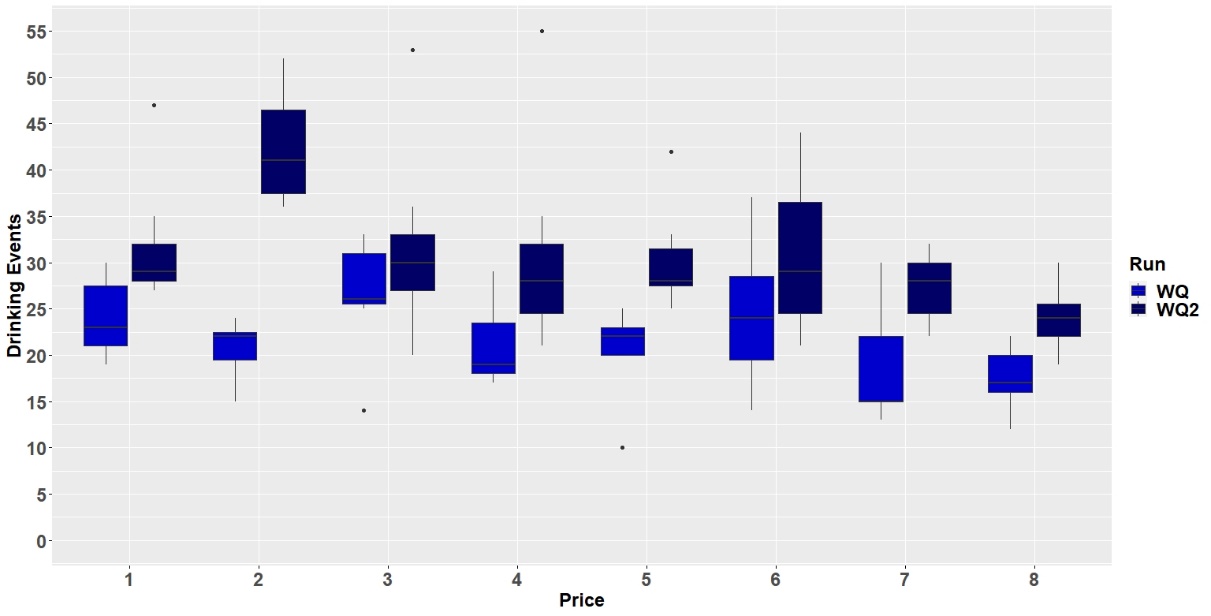


**Fig S1.** Comparison of drinking events (W = water, Q = quinine).

Pre-Test

For a successful consumer demand test, it is necessary that the animals have learned the task to prevent misinterpretation. If animals have not learned to work for access to certain goods and therefore work less, this could be falsely interpreted as a low motivation. Our pre-tests for our consumer demand test served to find the adequate method to train the mice to work (make nosepokes within the IntelliCage corner) for access to liquids.

Animals and housing conditions

For the pre-tests 11 female C57BL/6J mice (Charles River Sulzfeld, Germany) were used. To ensure maximum genetic and epigenetic independency between individuals, all mice had different mothers and foster mothers. The mice arrived at the institute at an age of 21 days. Before being subjected to the consumer demand test, the mice were used as experimental subjects in a home-cage based preference test in which various liquids were available for selection in order to generate a ranking. For the preference test the home-cage based and automated test setup presented here was also used. Accordingly, the mice were already familiar with the setup before the pre-test for the consumer demand test started. Within the home-cage nesting and bedding material, two red houses, four wooden bars to chew on, one Plexiglas handling tube and food ad libitum were available. All mice were handled by the tunnel method. During the pre-test, the mice were 17 to 21 months old. During this time, two mice had to be killed due to health issues unrelated to the ongoing experiment. Prior to the pre-test, all mice showed fur trimming behavior.

For testing within the IntelliCage (IC) system, it is necessary to implant radio frequency identification (RFID) transponders. These transponders were placed subcutaneously in the neck region of the mice under isoflurane anesthesia. Two hours before implantation, the mice received an analgesic (meloxicam, 1mg/kg, Meloxidyl by CEVA) orally. The mice received the transponders (Planet ID, FDX-B, ISO 11784/85) under isoflurane anesthesia (induction of anesthesia: 4l/min 4%; maintenance of anesthesia: 1l/min 1-2%) at an age of 33 days. Since three mice lost their transponder, the procedure had to be repeated (at an age of 56 days). The pre-test were conducted within an automated and home-cage based test setup, which is described in detail in the main paper section.

Method and results of the pre-tests

The mice had to work for access to almond milk in the working corner in the first three runs (AW 1, AW 2, AW 3), while water was freely available in the free corner. For access to almond milk, the mice had to make more nosepokes every day, except for run AW 2, when the mice had to perform four nosepokes each of the first three days (Table S1). When the required nosepoke number was made, the mice had 10 seconds to drink. After further 5 seconds an airpuff (5 bar for one second) occurred, should the mouse not have left the corner. This was to prevent individual mice from spending too much time in the corner (During the main consumer demand test, the airpuff was not used).

Over time, as the price increased, fewer mice made the necessary number of nosepokes, and there was a dependence between the maximum paid price and run (p < 0,0001, n = 11, figure S2). However, it was not clear whether the motivation to work for access to almond milk decreased with increasing price or whether the operant task was not learned.

Since we assume that almond milk was preferred by the mice over water, we examined the drinking events in the working corner (almond milk) and free corner (water) of the first day of the three different runs. Drinking events were defined as visits in which the mice made the required nosepoke number and drank. The number of the drinking events (logarithm of drinking events) were used as the outcome in a linear mixed-effects model. In this model, the three runs were defined as runs and used as a fixed effect (factor with three levels). In addition, the type of corner (factor with two levels: working corner versus free corner) and the interaction of type of corner and run were used also as fixed effects. To allow for the interpretation of the p-values of the main effects even with an interaction in the mode, we used sum-contrasts for run and type of corner. The runs nested in animals were set as a random effect. With the log-transformations of drinking events, we obtained normally distributed residuals. Model assumptions were inspected visually by Q-Q plots and by considering the variance homogeneity of the residuals versus the fitted model.

The data analysis showed that run (linear mixed-effects model: F-value = 12.18, p < 0.0001), corner (linear mixed-effects model: F-value = 35.0, p < 0.0001) and also the interaction (linear mixed-effects model Run:Corner: F-value = 64.37, p < 0.0001) had an influence on the drinking events. Post-hoc analysis (P package emmeans) showed that more almond milk was drunk in AW1 (post-hoc analysis: estimate = -3.48, SE = 0.33, df = 30, t.ratio = -10.71, p < 0.0001), more water in AW2 (post-hoc analysis: estimate = 1.68, SE = 0.33, df = 30, t.ratio = 5.175, p < 0.001) and more almond milk again in AW3 (post-hoc analysis: estimate = -1.53, SE = 0.33, df = 30, t.ratio = -7.71, p < 0.001).

We decided to change the training because we assumed that the operant task was not learned. Instead of working for access to a preferred liquid (almond milk), the mice had to work for access to water in the working corner to avoid drinking a bitter tasting liquid in the free corner (1.3 mM quinine hydrochloride dihydrate). To access water the mice had to perform one nosepoke more each day starting with one nosepoke on day one (run WQ). After 16 days, run WQ ended. All mice made up to 16 nosepokes for access to water in the working corner. This result confirmed that the operant task was learned by all mice. One more run followed in which the mice had to work again for access to almond milk (run AW) and one run in which the mice had to work for access to water to avoid drinking a sour-tasting liquid in the free corner (10 mM HCl, run WH). These runs were intended for identification of possible necessary adaptations but no further adjustments were needed. The results showed that the maximum price paid depended on the liquid (paid price analysis: n = 10, p < 0.0001, figure S3). This concluded the pre-test, and we proceeded with the main consumer demand test.

**Table S1** First three runs of the pre-test. The mice had to work for access to almond milk (A) in each run. Water (W) was available in the free corner. The daily required nosepoke number (RNN) was different in the three runs.

| **Day** | **RNN Run AW 1** | **RNN Run AW 2** | **RNN Run AW 3** |
| --- | --- | --- | --- |
| 1 | 1 | 4 | 1 |
| 2 | 2 | 4 | 2 |
| 3 | 4 | 4 | 3 |
| 4 | 8 | 5 | 4 |
| 5 | 12 | 6 | 5 |
| 6 |  | 7 | 6 |
| 7 |  | 8 | 7 |
| 8 |  | 9 | 8 |
| 9 |  |  | 9 |
| 10 |  |  | 10 |
| 11 |  |  | 11 |
| 12 |  |  | 12 |


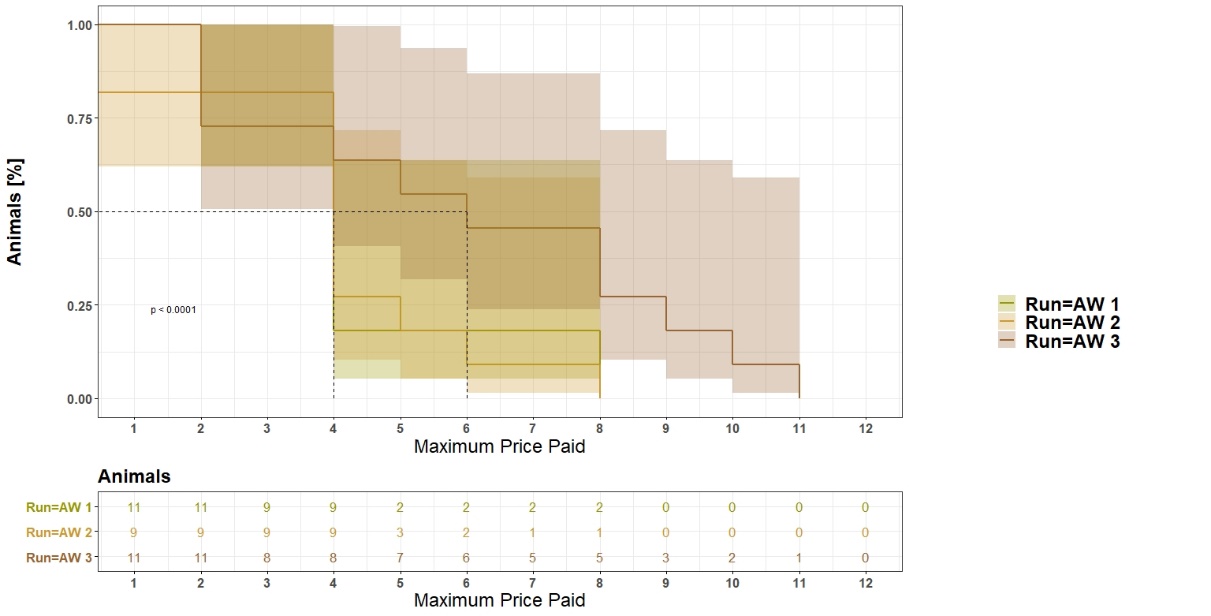


**Fig. S2** Price paid in the first three runs with almond milk (A) in the working corner and water (W) in the free corner.


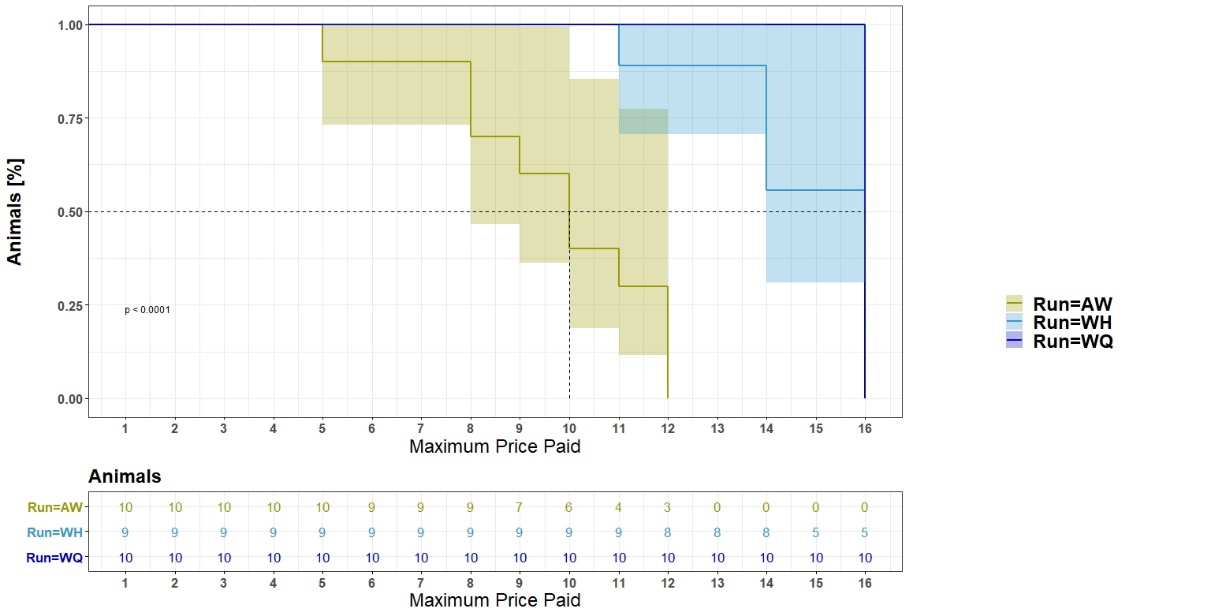


**Fig. S3** Paid price of three runs with different liquids to work for. First run: water (W) in the working corner and quinine (Q) in the free corner. Second run: almond milk (A) in the working corner and water in the free corner, Third run: water in the working corner and HCl (H) in the free corner.

Discussion

To perform a consumer demand test, it is essential that mice learned the operant task, otherwise misinterpretation may occur as our pre-test demonstrated. In our first runs during the pre-test, the mice had to work for access to almond milk, while water was available for the price of one nosepoke. With increasing price, fewer mice made the required nosepoke number to gain access to almond milk. During the first run (AW1), almond milk was preferred over water for the price of one nosepoke for both liquids which is in accordance with another study in our lab (unpublished data). The decreasing participation might show a low motivation to work for almond milk.

In the second run (AW2), the mice had to make four nosepokes on the first day for almond milk and one nosepoke for water, which resulted, contrary to our expectation, in more water being consumed than almond milk. This indicated the operant task was not learned. Therefore, we changed our strategy to train the mice in the operant task: The mice had to work for access to water, while they had access to quinine for the price of one nosepoke (run WQ). All mice made up to 16 nosepokes. This ensured the mice learned the operant task.

In summary, the training was more successful when the mice were asked to work to avoid what they perceived to be a negative stimulus. Therefore, successful training required the use of the right stimulus. For this reason, we started with the WQ run in the subsequent consumer demand test.
